# A Magnetic Tether System to Investigate Visual and Olfactory Mediated Flight Control in Drosophila

**DOI:** 10.3791/1063

**Published:** 2008-11-21

**Authors:** Brian J. Duistermars, Mark A. Frye

**Affiliations:** Department of Physiological Science, University of California, Los Angeles

## Abstract

It has been clear for many years that insects use visual cues to stabilize their heading in a wind stream. Many animals track odors carried in the wind. As such, visual stabilization of upwind tracking directly aids in odor tracking. But do olfactory signals directly influence visual tracking behavior independently from wind cues? Also, the recent deluge of research on the neurophysiology and neurobehavioral genetics of olfaction in Drosophila has motivated ever more technically sophisticated and quantitative behavioral assays. Here, we modified a magnetic tether system originally devised for vision experiments by equipping the arena with narrow laminar flow odor plumes. A fly is glued to a small steel pin and suspended in a magnetic field that enables it to yaw freely. Small diameter food odor plumes are directed downward over the fly s head, eliciting stable tracking by a hungry fly. Here we focus on the critical mechanics of tethering, aligning the magnets, devising the odor plume, and confirming stable odor tracking.

**Figure:**
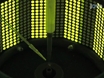


## Protocol

### Introduction

The OMT is an adaptation of a magnetic tether system [1] designed to incorporate a “virtual plume simulator”. The following protocols will explain how to properly tether flies (Part 1) and provide strategies for setting up the magnets (Part 2) and mass-flow regulated odor delivery system (Part3). The information described here is optimized for this particular system and may vary for components with other technical specifications.

### Part 1: Tethering flies

The steps for tethering described here are similar to many previous studies [2] but adapted for use in an OMT [3]. Proper tethering is crucial to ensure robust and repeatable experimental trials. The importance of careful tethering can not be overstated.

1. Collect flies 4-6 days post eclosion and starve them for 4-6 hours. Place a kimwipe moistened with water in the foodless vial to prevent desiccation.
2. Transfer a batch of flies from a bottle to a small vial and insert it into a brass block on a Peltier cooling stage set at approximately 4 degrees Celsius. The proper temperature anesthetizes the flies within approximately 30 seconds.
3. Sort the flies on the cold surface of the cooling stage. A damp Kimwipe ensures constant cooling and moisture wicking from the cuticle. Select the largest females for tethering.
4. Using a fine brush, push a single fly into position in the custom-built sarcophagus shaped to accommodate a single wild type female fruit fly.
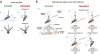
Figure 1.Please click here to see a larger version of the the figure.
5. We position a minutien pin (Fines Science Tools) with a magnetic rod mounted on a micromanipulator. The pin is to be aligned such that the blunt end touches the dorsal thorax just behind the head between the two wings. The pin should be perfectly vertical when viewed head-on toward the eyes. Even a small amount of ‘roll’ between the fly and pin can not be tolerated. The side-view angle between the pin and the long axis of the body should be at a nose-up angle of 30 degrees relative to the horizon (Fig. 1). Note: For optimal operation in the magnet tether, the pins may need to be trimmed to shorter lengths. This will depend entirely on the distance between the suspension magnets.
6. Once the minutien pin is aligned, use a thin steel wire to apply a small drop of UV-activated glue (ElectroLite Corp) to the blunt end of the minutien pin.
7. Manipulate the pin so the glue droplet touches the fly’s thorax just above the head. With the proper drop size, the glue should anneal and flow from the pin onto the thorax. Be careful not to use too much glue.
8. Cure the glue with two 20 second bursts of UV light.
9. Place the fixed fly aside and repeat. Slice small squares of Kimwipe and with forceps give one to each tethered fly to restrain flight.
10. After one hour of recovery, the tethered flies are ready for experiments.
11. Improper gluing may hinder a fly’s ability to perform in the magnet arena. Every fly should be checked for smooth rotation at the start of every experiment to ensure the flies are properly glued. This can be accomplished by rotating a striped pattern with a sufficiently large spatial period to elicit strong optomotor responses [4]. If the fly is glued at the wrong pitch angle or tilted on the pin with respect to the roll axis, the fly may (i) not spin at all, (ii) have a restricted range of motion, or (iii) rotate at inconsistent velocity. In the above cases the fly should be discarded and replaced with a new one. If this solves the problem it was most likely a case of improper gluing. (if replacing the fly does not solve the problem, see Part 2.4)

### Part 2: Aligning the magnets

Aligning the magnets (Rare-earth-magnets.com) properly is necessary for the fly to achieve a smooth 360 degree range of motion. Follow these steps to properly align the magnets.

1. The lower ring magnets are held horizontally by a plastic sleeve and placed directly on top of a clear vacuum chamber (Fig. 1; described further in part 3). The upper rod magnet is fixed vertically approximately 3/4" above the lower magnets.
2. Roughly align the upper and lower magnets visually by placing the upper rod magnet directly above the center point of the lower ring magnets. It is useful to attach the upper magnet to a micromanipulator (Siskiyou Inc.) for fine scale adjustment.
3. Epoxy a V-jewel bearing (Small Parts) on the bottom surface of the upper magnet to minimize rotational friction and standardize fly positioning.
4. Place a fly in the arena and adjust the horizontal and vertical position of the upper magnet until the fly can smoothly and steadily rotate 360 degrees in the yaw axis. Every fly should be checked for smooth rotation at the start of every experiment to ensure the magnets are properly aligned and that individual minutien pins are not damaged (see Part 1.11). If the magnets are not properly aligned the fly may (i) not spin at all (ii) have a restricted range of motion (iii) rotate at inconsistent velocities. If the problem is solved by replacing the fly with a new one, it was most likely due to a gluing problem (see Part 1). If these problems exist for every fly, it is likely an issue with the alignment of the magnets and further adjustment will be required.
5. Warning: while playing with rare earth magnets can be fun, they are very dangerous. Misuse results in damaged equipment and/or minor bodily harm. Exercise extreme caution with these tempting yet easily underestimated magnets.

### Part 3: Odor Delivery

1. The odor delivery system is based on one described previously [5] and is the toughest part to optimize (Fig. 1). It requires a lot of patience and trial-and-error. Follow these steps to achieve stable odor tracking.
2. The initial set-up of the odor system requires running narrow gauge Teflon tubing (Small Parts) from the gas multiplexer (Sable Systems International) to the custom built water/odor vials. The outlet of these vials connects directly to the hypodermic port tubes using the same Teflon tubing.
3. A clear acrylic vacuum chamber is positioned under the arena to support the magnets. We use a 4mm glass tube mounted below the fly connected to the vacuum chamber to bring the vacuum opening closer to the fly and reduce the diameter of the opening.
4. Place a tethered fly into the arena using forceps.
5. Turn on an attractive odor (apple cider vinegar works well), and visually verify that the fly tracks the odor. If the fly appears to have no preference for the odor, follow the next three steps. This may require several flies, and several trials.
6. Adjust the position of the odor port until the fly appears to track the odor. We mounted the odor ports on micromanipulators (Siskiyou Inc.) and positioned 4mm dorsal and 3mm anterior to the fly’s head.
7. Adjust the mass flow rate until the fly appears to track the odor. Our settings are 7ml/min (Sable Systems International MFC-4).
8. Adjust the flow rate of the vacuum until the fly appears to track the odor. We set the vacuum flow rate at 13l/min using a flow regulator (Cole Parmer). Note: Building vacuum supply lines may fluctuate over time causing fluctuations in plume structure. It may be useful to connect an external vacuum pump to the flow regulator for constant vacuum flow conditions.
9. Once the position of the port and the flow rates are set, periodically switch between delivering water vapor and odor vapor using a switchable using a gas multiplexer and visually verify that the fly only tracks the odor when the odor is on. Occasionally, residual odor inside the odor port can elicit odor tracking when the odor is turned off. This can be eliminated without removing the properly positioned odor port by filling the hypodermic tubes with ethanol and collecting it on the delivery end with a Kimwipe. Using compressed air, blow any remaining ethanol out and resume experiments.

### Representative Results:

When these steps are followed correctly two things should be observed. First, under constant rotating visual stimuli a tethered fly placed in the OMT should rotate smoothly 360 degrees in the yaw axis. “Jerky” turns (called saccades) are characteristic of spontaneous turning behavior in the absence of constant visual stimuli. If flies can apparently not rotate smoothly, improper gluing (see Part 1) and misaligned magnets may be to blame (see Part 2). Second, when the odor is turned on, the fly should head in the direction of the odor. When the odor is turned off, the fly should saccade spontaneously with no preference for any single position in the arena. If flies do not actively track the odor plume, the position of the odor port, the air flow rate and/or the vacuum flow rate may need adjustment (see Part 3).

## Discussion

Tethering flies and eliciting stable odor tracking in this arena is relatively straightforward. It requires practice, patience and trial and error. Many parts of this arena could be further optimized or substituted to yield variations in experimental potential. For example, newer versions of the vacuum chamber are much smaller and allow more space below the arena. Also, the strength and type of rare-earth magnets can be varied slightly and additional odor ports could be added to increase the number of odor types and/or concentrations utilized in experiments. We note that any variations in equipment will require tailored optimization. As described here, the OMTA allows users to test the effect of visual cues on odor localization. A wide variety of visual stimuli can be presented with the LED panel system and when coupled with the versatility and expandability of the odor delivery system the system offers the potential to explore visuo-olfactory integration at many levels. This system also adds to the increasing number of quantitative behavioral assays for Drosophila, particularly for characterizing the behavior of flying adults.
